# Diagnostic evaluation to identify infection-attributable stillbirth

**DOI:** 10.1038/s41372-025-02253-w

**Published:** 2025-03-06

**Authors:** Sarah A. Coggins, Jourdan E. Triebwasser, Karen M. Puopolo

**Affiliations:** 1 Division of Neonatology, Children’s Hospital of Philadelphia, Philadelphia, PA USA; 2 Department of Pediatrics, University of Pennsylvania, Philadelphia, PA USA; 3 Clinical Futures, Children’s Hospital of Philadelphia, Philadelphia, PA USA; 4 Division of Maternal-Fetal Medicine, Department of Obstetrics and Gynecology, University of Michigan, Ann Arbor, MI USA

**Keywords:** Epidemiology, Infection

## Abstract

**Objective:**

To characterize stillbirth evaluations, including the frequency and yield of investigations for infections causing stillbirth.

**Study design:**

Retrospective cohort of stillbirths at three university-affiliated perinatal centers from 2017 to 2022. The primary outcome was adherence to American College of Obstetrics and Gynecology core stillbirth evaluation recommendations (placental pathology, fetal autopsy, and fetal genetic testing). We further characterized the prevalence and yield of specific testing to evaluate for infection-attributable stillbirth etiologies.

**Results:**

The cohort included 399 stillbirths. Placental pathology was performed in 387 cases (97.0%), fetal genetic testing in 163 (40.9%), and fetal autopsy in 126 (31.6%). Fetal bacterial cultures were obtained in 73 (18.2%) cases; potential pathogens were isolated in 21/73 (28.8%). Viral testing was sent infrequently, with variable yield. Six stillbirths had infections identified as probable etiologies.

**Conclusions:**

Adherence to core stillbirth evaluation recommendations was poor, and infection testing was infrequent. Infection-attributable stillbirth prevalence may be underestimated.

## Introduction

Stillbirth is a devastating pregnancy outcome, with potential risks for future pregnancy depending on the etiology. An estimated 2.6 million pregnancies annually result in stillbirth, corresponding to an incidence of 18.4 stillbirths per 1000 total births globally. [1] In the United States (U.S.) during 2021, there were 21,105 estimated stillbirths at ≥20 weeks of gestation (5.73 stillbirths per 1000 total births). [2] Maternal risk factors for stillbirth are well-described and include disparity in pregnancy outcomes due to systemic and structural racism, maternal age at youngest and oldest extremes, lower maternal educational attainment, obesity, pre-pregnancy hypertension, maternal vascular or autoimmune diseases, nulliparity, and prior preterm birth or stillbirth. [3–5] Fetal risk factors may include congenital genetic or structural anomalies. [5] Despite these known risk factors, contemporary U.S. surveillance data find that the underlying etiology is unknown in 31% of stillbirths [6], similar to estimates from Australia (29%) [7] and the United Kingdom (46%). [8]

Identifying the cause of an individual stillbirth may provide closure for families, identify maternal conditions whose treatment may optimize future pregnancy outcomes, and improve future counseling and surveillance. Infections are a potentially preventable but poorly-understood stillbirth etiology. The utility and cost-effectiveness of routine evaluations for infection following stillbirth are debated and abnormal testing for infection may not prove causality. [5, 9–13] Consequently, the reported incidence of infection-attributable stillbirth in high-income countries varies substantially, ranging from 1 to 25%. [6, 12–14] Mechanisms of infection-mediated stillbirth vary and include direct fetal infection, infection-mediated placental dysfunction, severe maternal illness, and/or preterm labor or preterm premature rupture of membranes (PPROM). [5]

The American College of Obstetricians and Gynecologists (ACOG) recommends diagnostic evaluations following stillbirth to identify a probable etiology. [15] Our objective was to characterize the core recommended components of stillbirth evaluation in a large healthcare system, with specific attention to the frequency and yield of additional investigations for infections causing stillbirth.

## Materials and methods

### Study design and study population

Retrospective cohort of stillbirths occurring during calendar years 2017-2022 at three perinatal centers affiliated with the University of Pennsylvania Health System. Two centers are urban academic tertiary care centers, while the third is community-based. We identified all stillbirths, specifically coded within the electronic health record (EHR) and defined as births ≥20 0/7 weeks gestation with Apgar scores of 0, 0, and 0 at 1, 5, and 10 min after birth. We excluded stillbirths following pregnancy termination from the primary cohort. Participant clinical data were collected via manual chart review of the health system’s common EHR. This study was approved by the Institutional Review Board at the University of Pennsylvania and granted a waiver of informed consent.

### Data collection

Maternal characteristics included age, race (self-reported, acknowledged as a social construct, and collected to assess for disparity in stillbirth evaluation) [2], and maternal medical diagnoses. Obstetric history included gravidity, parity, history of recurrent ( ≥ 2) spontaneous abortions before 20 weeks gestation, history of prior stillbirth, receipt of any prenatal care, use of assisted reproductive technology, prenatal diagnosis of congenital anomalies, and presence of specific maternal medical conditions (e.g., diabetes, autoimmune disease) and pregnancy complications (e.g., preeclampsia, placental abruption, cholestasis of pregnancy) potentially associated with stillbirth. Gestational age (GA) at the time of stillbirth was categorized into groups (20–23, 24–27, 28–33, 34–36, and ≥37 weeks). Maternal laboratory, pathology, and imaging data were collected as well as data on postnatal fetal evaluations.

### Statistical analysis

The primary objective was to describe patterns of diagnostic testing obtained in the course of stillbirth evaluation. Core elements of stillbirth evaluation were identified via the Care Consensus for Management of Stillbirth, published in March 2020 by ACOG and Society for Maternal-Fetal Medicine (SMFM) [15]. We assessed for associations between ACOG core components of stillbirth evaluation (placental pathology, fetal autopsy, and fetal genetics) with the following factors: birth hospital, maternal race, receipt of any prenatal care, history of recurrent spontaneous abortion or prior stillbirth, GA category, and maternal/fetal conditions complicating pregnancy (preeclampsia, placental abruption, obstetric diagnosis of chorioamnionitis, fetal growth restriction, and prenatally-diagnosed congenital anomalies). We further analyzed the frequency and yield of maternal and fetal testing targeted at potential infections causing stillbirth. We analyzed factors associated with maternal and fetal infection testing, including maternal factors complicating pregnancy (pre- or periviable preterm labor or PPROM > 18 h, preeclampsia, or placental abruption), fetal factors (GA category, history of fetal growth restriction, prenatally-diagnosed anomalies), and delivery factors (delivery mode, obstetric diagnosis of chorioamnionitis). The primary cohort included all eligible stillbirths. Characteristics of stillborn infants born following pregnancy termination are described in Supplemental Table 1, which we included given an a priori anticipation that infectious testing may have been more prevalent in this population, and that some terminations may have otherwise resulted in stillbirth (particularly those complicated by fetal anomalies or congenital infections). We summarized demographic and clinical data using standard descriptive statistics. To assess associations between categorical variables, we performed Fisher’s exact test or Pearson’s chi-square test, as appropriate. We used the Cochran-Armitage test to measure trends across categorical variables. Two-sided p-values < 0.05 were considered statistically significant. All analyses were performed using Stata v.17 (College Station, TX, USA).

## Results

### Study population

During the study period, we identified 432 stillbirths and 74,223 live births at ≥20 weeks gestation across three perinatal centers. There were 354 stillbirths (88.7%) from singleton pregnancies, 42 (10.5%) from twin pregnancies, and three (0.3%) from triplet pregnancies. Thirty-three stillbirths occurred following pregnancy termination, were excluded, and are described in Supplemental Table 1. The primary analytic cohort therefore included 399 stillborn fetuses among 389 pregnancies for an observed stillbirth incidence of 5.3 per 1000 total births.

Maternal characteristics are presented in Table 1. Median maternal age at stillbirth was 29 years (interquartile range [IQR] 25, 34), with median GA of 28 5/7 weeks (IQR, 23 0/7, 35 1/7). Genitourinary infections (18.5%, primarily urinary tract infections), preeclampsia (16.4%), and placental abruption (15.4%) were the most commonly-documented pregnancy-related complications. Maternal respiratory infections during pregnancy occurred prior to 28 (7.2%) stillbirths; 20 were due to COVID-19, with only 3/20 parturients having received a COVID-19 vaccine. Ultrasound-based estimated fetal weight <10^th^ percentile was present in 71 (17.8%) stillbirths, while prenatally-diagnosed congenital anomalies were present in 84 (21.1%).

**Table 1:** Maternal demographics and clinical characteristics.

	N = 389^a^
Maternal Characteristics at Delivery (n, %)	
Maternal age in years, median (IQR)	29 (25, 34)
Maternal race	
Asian	23 (5.9)
Black	211 (54.2)
Native American or American Indian	2 (0.5)
Native Hawaiian or Pacific Islander	2 (0.5)
Unknown	55 (14.1)
White	96 (24.7)
Gestational age weeks, median (IQR)	28 5/7 (23 0/7, 35 1/7)
Maternal Medical History, n (%)	
Pre-pregnancy body mass index in kg/m^2^, median (IQR)^b^	26.6 (23.2–33.0)
Chronic hypertension	46 (11.8)
Diabetes	30 (7.7)
Type 1	2 (0.1)
Type 2	16 (4.1)
Gestational	12 (3.1)
Lupus	4 (1.0)
Other autoimmune disease	8 (2.1)
History of thromboembolism	1 (0.3)
Epilepsy	1 (0.3)
Cardiac disease	9 (2.3)
Maternal Obstetric History, n (%)	
Gravidity (median, IQR)	3 (1, 4)
Parity (median, IQR)	1 (0, 2)
Current multiple gestation pregnancy	35 (9.0)
History of ≥2 recurrent spontaneous abortions	42 (10.8)
History of prior stillbirth	26 (6.7)
History of maternal hereditary diagnosis^d^	3 (0.7)
Consanguineous pairing	5 (1.3)
Characteristics of Pregnancies ending in Stillbirth, n (%)	
Any prenatal care	362 (93.1)
IVF or IUI-assisted pregnancy^c^	15 (3.9)
Prenatally-diagnosed congenital anomalies	84 (21.1)
Fetal growth restriction <10^th^ percentile	71 (18.3)
Diagnoses complicating current pregnancy	
Preeclampsia	64 (16.4)
Placental abruption	60 (15.4)
Obstetric diagnosis of chorioamnionitis	57 (14.7)
Abdominal trauma	5 (1.3)
Cholestasis of pregnancy	0 (0)
Respiratory infection	28 (7.2)
Genitourinary infection	72 (18.5)
Opiate use disorder	14 (3.6)
Alcohol use disorder	2 (0.5)
Use of illicit (non-opiate) drugs	18 (4.6)
Cesarean delivery	56 (14%)

^a^Pregnancy-level summary of maternal data

^b^Data available for 285/399 (71%) of parturients

^c^IVF, in vitro fertilization; IUI, intrauterine insemination

^d^Included osteogenesis imperfecta, autosomal dominant cardiomyopathy, hemoglobin E disease with beta-thalassemia

Stillbirths during multiple gestation pregnancies occurred at significantly lower GA compared to singleton stillbirths (median 23 0/7 weeks vs 29 4/7 weeks, p < 0.001) and were significantly more likely to deliver via Cesarean (24.4% vs 12.7%, p = 0.04). (Supplemental Table 2)

### Characteristics of stillbirth evaluations

The prevalence of ACOG-recommended stillbirth evaluation elements are presented in Table 2. Among Grade 1 A elements (placental pathology, fetal autopsy, and fetal genetic testing), only 97 (24.3%) stillbirths had all three investigations performed. Placental pathology was performed in the majority (97.0%) of stillbirths (Table 2). Fetal genetic testing was obtained in 163 (40.9%) stillbirths and yielded abnormal results (e.g., reported pathogenic variants or variants of unknown significance) in 18 cases (11.0%). Karyotype analysis was attempted in 115 stillbirths but no result was obtained in 25/115 (21.7%) cases. Whole exome sequencing was performed in four stillbirths, with abnormal results in two. Fetal autopsy was obtained in 31.6% of stillbirths. Additional maternal elements of the stillbirth evaluation are shown in Table 2. There were no differences in the frequency of placental pathology, fetal autopsy, or fetal genetic evaluations when comparing stillbirths occurring during singleton vs multiple gestation pregnancies. (Supplemental Table 2). However, karyotype analysis was performed significantly more frequently among stillbirths occurring in singleton compared to multiple gestation pregnancies (30.8% vs 13.3%, p = 0.01).

**Table 2:** Frequency of ACOG-recommended stillbirth evaluation completion.

Evaluation	Frequency of Completion, n (%)	Comments
Fetal-Placental Evaluation (N = 399)		
Placental pathology	387 (97.0)	ACOG Grade 1 A recommendation
Documented gross fetal examination	252 (63.2)	ACOG Grade 1 C recommendation
Genetic analysis	163 (40.9)	ACOG Grade 1 A recommendation
Karyotype	115 (28.8)	
Microarray	117 (29.3)	
Both karyotype and microarray	70 (17.5)	
Whole exome sequencing	4 (1.0)	
Fetal autopsy	126 (31.6)	ACOG Grade 1 A recommendation
Maternal Evaluation (N = 389)		
Antiphospholipid antibody testing^a^	222 (57.1)	Recommended in absence of anomaly or suspected infection
Kleihauer-Betke testing	199 (51.2)	Recommended in absence of anomaly or suspected infection
Toxicology screen	197 (50.6)	Recommended in selected cases
Glucose screening		Recommended in selected cases
Glucose tolerance testing available^b^	162 (41.6)	
Hemoglobin A1c	304 (78.1)	

^a^Includes lupus anticoagulant, anticardiolipin antibodies, and/or beta-2 glycoprotein antibodies

^b^At minimum, available results of 1-hour glucose tolerance testing

We compared stillbirth evaluations occurring before (2017–2019) and after (2020–2022) publication of the updated SMFM guidance (Supplemental Table 3). There was significantly more fetal genetic testing obtained post- compared to pre-guidance, with no differences in the proportions of stillbirths evaluated with placental pathology or fetal autopsy. There was no change in the proportion of stillbirths tested for maternal or fetal infection following guideline publication.

Fetal autopsy rates differed significantly by gestational age, increasing as GA category increased (Supplemental Table 4). Fetal autopsy was performed for 20/120 (16.7%) stillborn infants born 20–23 weeks GA, compared to 23/55 (41.8%) infants born ≥37 weeks GA (p = 0.001). Fetal genetic testing differed significantly by GA category without linear trend; testing occurred most frequently among stillbirths at 24–27 weeks GA (41/69, 59.4%) and ≥37 weeks GA (27/55, 49.1%), and least frequently at 20-23 weeks GA (33/120, 27.5%) (p < 0.001).

Stillbirths occurring at the community-based perinatal center, compared to the two tertiary academic centers, were significantly less likely to have fetal autopsies (3.5% [3/85] vs. 44.6% [123/314], p = 0.001) and fetal genetic testing (25.9% [22/85] vs 44.6% [140/314], p = 0.002). Fetal genetic testing, but not fetal autopsy, was more commonly performed among stillbirths with fetal growth restriction (41/72 [56.9%] vs 121/327 [37.0%], p = 0.002) or prenatally-diagnosed congenital anomalies (56/86 [65.1%] vs 105/310 [33.9%], p < 0.001). There were no associations of maternal race, receipt of any prenatal care, history of recurrent spontaneous abortion, or history of stillbirth with the performance of placental pathology, fetal autopsy, or fetal genetics.

### Prevalence and yield of adjunctive testing for infections

The prevalence and yield of specific testing for infection are presented in Table 3. Maternal syphilis testing results were available for almost all parturients (383/389, 98.4%). In contrast, testing for other infections was obtained in less than half of stillbirths. Fetal bacterial cultures from any site were performed in 73/389 (18.3%). These were usually surface, blood, or tissue (lung, liver, spleen) cultures obtained at the pathologist’s discretion during fetal autopsy. Among all cultured, 21/73 (28.7%) exhibited culture growth that was considered potentially pathogenic and not due to postmortem contamination. Identified organisms included *E. coli* (10/21, 47.6%), *Enterococcus faecalis* (4/21, 19.0%), and group B *Streptococcus* (3/21, 14.3%). Most fetal bacterial cultures (58/73, 79.5%) were obtained during a fetal autopsy, but cultures were done in less than half (58/126, 46.0%) of all fetal autopsies. Fetal/placental viral and parasitic testing was infrequently obtained, and generally during fetal autopsy. Polymerase chain reaction (PCR) testing of fetal tissue (generally liver or spleen, at the pathologist’s discretion) for cytomegalovirus, *Toxoplasma gondii*, and parvovirus occurred among 7.8%, 3.3%, and 1.0% of all stillbirths, respectively. Of those, cytomegalovirus PCR was positive in 2 cases (6.5%), *Toxoplasma* was positive in 1 case (25%), and parvovirus was negative in all cases. Maternal serologic testing was more frequently obtained than fetal PCR, with cytomegalovirus, *Toxoplasma*, and parvovirus serologies obtained among 16.7%, 14.6%, and 37.8% of parturients, respectively. Cytomegalovirus and parvovirus IgG were positive in over half of tested individuals; IgM was positive in 1.5% and 0.7%, respectively.

**Table 3:** Prevalence and yield of adjunctive infectious testing.

Test	Prevalence, n (%)	Abnormal results among those tested, n (%)
Maternal testing (N = 389)		
Maternal infectious testing		
Syphilis^a^	383 (98.4)	2 (0.5)
Parvovirus IgG	146 (37.5)	82 (56.2)
Parvovirus IgM	146 (37.5)	2 (1.4)
Cytomegalovirus IgG	65 (16.7)	39 (60.0)
Cytomegalovirus IgM	65 (16.7)	1 (1.5)
Toxoplasma IgM	56 (14.4)	0 (0)
Other maternal testing		
Antiphospholipid antibody screening	221 (56.8)	28 (12.7)
Toxicology screening	197 (50.6)	42 (21.3)
Kleihauer-Betke testing	196 (50.4)	17 (8.7)
Fetal testing (N = 399)		
Fetal infectious testing		
Bacterial culture	73 (18.3)	21 (28.8)
Cytomegalovirus PCR	31 (7.8)	2 (6.5)
Parvovirus PCR	13 (3.3)	0 (0)
Toxoplasma PCR	4 (1.0)	1 (25.0)

^a^Includes treponemal and/or non-treponemal testing

Among stillbirths occurring during singleton vs. multiple gestation pregnancies, there was no difference in the frequency of evaluation by fetal bacterial culture, fetal/placental cytomegalovirus PCR, or fetal/placental parvovirus PCR testing. However, maternal serologic testing for cytomegalovirus and for parvovirus occurred significantly more frequently among singleton compared to multiple gestation pregnancies (respectively, 18.1% vs 2.2%, p = 0.004; and 41.2% vs 4.4%, p < 0.001). (Supplemental Table 2)

We next analyzed potential associations between prespecified clinical factors and maternal or fetal testing for infection. An obstetric diagnosis of chorioamnionitis was the only factor associated with collection of fetal bacterial cultures, with cultures obtained in 38.6% (22/57) of cases with a diagnosis of chorioamnionitis and 15.0% (51/341) of those without (p < 0.001). Fetal cytomegalovirus testing was associated with stillbirth in the setting of fetal growth restriction (10/72 [13.9%] vs. 21/327 [6.4%], p = 0.03); no clinical factors were associated with decisions to obtain fetal parvovirus testing. Maternal cytomegalovirus testing was associated with fetal growth restriction (25.0% vs 14.4%, p = 0.027) and prenatally-diagnosed congenital anomalies (27.9% vs 14.7%, p = 0.001).

### Probable infection-attributable stillbirths

After manual review of available clinical history and laboratory testing, we identified six of 399 stillbirths whose probable etiology was attributed to infection by maternal-fetal medicine physicians and/or pathologists. Congenital cytomegalovirus was the identified etiology in two stillbirths, both diagnosed primarily on characteristic inclusions found on autopsy. Congenital toxoplasmosis was identified on placental pathology in one stillbirth presenting with pathognomonic signs (growth restriction, cerebral calcifications, organomegaly). Two stillbirths were attributed to intrauterine infection due to GBS, in the setting of maternal GBS sepsis (first case) and positive GBS cultures in fetal lung and spleen (second case). A sixth case was attributed to inflammatory sequelae of *E. coli* infection, with positive fetal blood cultures recovered on autopsy.

## Discussion

In this retrospective cohort study, we reviewed the clinical course and postnatal evaluations of almost 400 pregnancies ending in stillbirth. Several important findings emerged relevant to evaluation for infections causing stillbirth. First, adherence to ACOG Grade 1 A stillbirth evaluation recommendations was variable: while placental pathology was completed in almost all cases, fetal autopsies and fetal genetic testing were performed in less than half of stillbirths. Second, updated national guidance on stillbirth evaluation resulted in an increase in the rate of genetic testing but other recommended evaluations were still lacking. Finally, targeted fetal and maternal testing for specific bacterial and viral pathogens occurred infrequently - yet the yield of such investigations was substantial when performed. Our findings suggest that the prevalence of infectious etiologies underlying stillbirth may be underestimated.

The Stillbirth Collaborative Research Network (SCRN) found that systematic stillbirth evaluations can identify the probable cause of fetal death in 60% of cases, and possible and probable causes in 76%. [14] The utility of specific tests was evaluated among an SCRN cohort of 512 stillbirths. [16] Placental pathology was deemed useful in 65% of cases, fetal autopsy in 42% and genetic testing in 12%, while serologic testing for parvovirus and syphilis less so ( < 1%). [16] Among 1025 stillbirths in the Netherlands, the most diagnostic utility was observed in placental pathology (96%), fetal autopsy (73%), and fetal genetics (29%). [17] Stepwise evaluation of 104 stillbirths identified probable etiologies in successively increasing frequency after considering clinical/laboratory history alone (24%), placental pathology (61%), and fetal autopsy (74%). [18] Assessing published evidence, the ACOG/SMFM stillbirth management 2020 consensus recommends placental pathology, fetal autopsy, fetal genetics, and a thorough maternal/obstetric history as core components of stillbirth evaluation. [15]

Despite available consensus recommendations, our study identified variable use of core stillbirth evaluation elements. These core elements may identify the stillbirth etiology (including infection-attributable etiologies) even without additional focused testing for infection, as occurred in at least 3 of 6 probable infection-attributable stillbirths in our study. Incomplete adherence to recommended practice was noted in 2014 U.S.-based population data that found fetal autopsy was not performed in 79% of stillbirths, while up to 31% had no placental pathology. [19] Barriers to completing stillbirth evaluation include a lack of perceived diagnostic utility, misconceptions about autopsy processes, parental cultural and religious beliefs, cost, and variable access to perinatal pathologists. [20, 21]^,^ We further identified variation in completion of stillbirth evaluation elements among singleton vs multiple gestation pregnancies; there was no difference in core component completion, but singleton pregnancies more frequently had genetic and maternal serologic testing for infection. While multiple gestation is in itself a risk factor for stillbirth, thorough evaluations following multiple gestation stillbirth still might identify an etiology relevant to future pregnancy care.

There are limited data informing the diagnostic efficiency of additional testing for maternal and fetal infection, despite data suggesting that infection may be associated with up to 25% of stillbirths in high-income countries (with even higher rates in low- and middle-income countries). [1, 22] A study utilizing bacterial 16S rRNA PCR analyses in cord blood found significantly higher bacterial burden and pathogen distribution among stillbirths compared to live births, acknowledging this method’s limitations in assigning causality. [23] Another study of 66 stillbirths with a probable or possible infectious etiology observed that all cases had evidence of abnormal inflammation or infection on placental pathology and/or fetal autopsy. [12] In this study, recognized pathogens—predominantly GBS, *E. coli*, and *Enterococcus* species—were isolated from fetal bacterial cultures obtained at autopsy. The incidence of GBS-attributable stillbirth alone is estimated at 0.04-0.9 per 1000 live births globally, accounting for 1% of stillbirths in high-income countries compared to 4% in low-income countries in Sub-Saharan Africa. [24, 25] The role of emerging or recrudescent infection may be underestimated if not measured. For example, despite increasing U.S. rates of congenital syphilis infection and an 8% global syphilis-attributable stillbirth rate, syphilis testing is inconsistent in the setting of stillbirth. [1, 26–28] Due to local regulations, maternal syphilis testing in our study was near-universal, in contrast to the 51% testing rate in a contemporary retrospective cohort of over 1,100 stillbirths in Indiana. [27]

Numerous viruses (e.g., cytomegalovirus, parvovirus, enteroviruses, human herpesvirus-6, and COVID-19) and parasites (*Toxoplasma gondii* and *Plasmodium falciparum)* have been implicated in stillbirth. [11, 29] Despite this, controversies surround the diagnostic efficiency and cost-effectiveness of routine testing for infection in stillbirth evaluation. The limitations of maternal serology testing are well-reported, with noted inefficiency in the absence of concordant clinical history or fetal anomalies. [13, 17] Our study confirmed this, with positive cytomegalovirus and parvovirus IgM results in only 1% of tested parturients. In contrast, direct fetal testing for infection was obtained less frequently in our stillbirth cohort, but among those tested, a substantial proportion yielded potentially meaningful abnormal findings. Fetal viral PCR was obtained infrequently ( < 10% of all stillbirths), yet abnormal results were identified and provided clear stillbirth etiologies for 6.5% of fetuses with cytomegalovirus testing and 25% of fetuses tested for *Toxoplasma gondii*. Among stillbirths for whom fetal bacterial cultures were obtained, approximately one-third isolated potentially pathogenic, non-contaminant organisms. It is likely that the clinicians in our study employed infection testing in history-targeted contexts, but the yields support the idea that expanded fetal bacterial and viral testing may enhance diagnoses of stillbirth etiology.

A more efficient approach to stillbirth evaluation that accounts for infectious etiologies may require a focus on fetal rather than maternal testing. Universal placental pathology and fetal autopsy would allow for progression to bacterial culture and/or viral PCR in all cases where pathologic examination suggests the presence of infection. Full fetal autopsy is not always performed due to clinical, social, or economic concerns, but targeted sampling can allow for limited fetal testing (cultures and viral PCRs) with important diagnostic yield. Ongoing critical evaluation of testing utility and cost-effectiveness is required, balancing potential diagnostic benefits with economic burdens of expanded testing for fetal infection.

Strengths of our study include manual chart review of almost 400 stillbirths occurring over a 6-year period, with detailed data collection regarding obstetric history, delivery course, and stillbirth evaluation. We acknowledge limitations. This retrospective review of clinical practice in our highly-resourced academic health system may not be generalizable to all settings. We could not retrospectively determine provider and/or family reasoning to obtain or defer specific stillbirth evaluation elements. Our primary objective was to characterize stillbirth evaluation, focusing on infectious components, and not to adjudicate the underlying probable and/or possible causes of fetal demise. Therefore, we cannot comment upon the distribution of stillbirth etiologies in this cohort, nor the association of any postnatal testing with the presumed stillbirth etiology.

## Conclusions

Our results suggest that local practices can be optimized to better identify potential infectious contributions to stillbirth. Adherence to ACOG Grade 1 A recommendations for core diagnostic testing combined with direct fetal bacterial, viral, and parasitic testing could result in the optimal etiologic capture. This, in turn could support preventative measures for both the individual patient and for the pregnant population and improve future pregnancy outcomes.

## Supplementary information

Supplemental Tables

## Data Availability

The dataset generated during and/or analysed during the current study is not publicly available.
