# Supplementary material for: Diagnostic evaluation to identify infection-attributable stillbirth
Source: J Perinatol. 2025 Mar 6;45(7):971–6. doi: 10.1038/s41372-025-02253-w (PMC12316590; doi:10.1038/s41372-025-02253-w)
Supplement: Supplementary file 1 — Supplemental Tables [file 41372_2025_2253_MOESM1_ESM.docx]

**Supplemental Table 1: Characteristics of Stillbirths Following Pregnancy Termination (N=33)**

| **Characteristic** | **n (%)** |
| --- | --- |
| Gestational age weeks, median (IQR) | 23 1/7 (22 2/7, 23 4/7) |
|  |  |
| Presence of fetal growth restriction | 10 (30.3) |
| Prenatally-diagnosed congenital anomalies | 26 (78.8) |
| Amniocentesis performed | 16 (48.5) |
|  |  |
| Reason for termination^1^ |  |
| Congenital anomalies | 19 (57.6) |
| Genetic disorder | 8 (24.2) |
| Infectious condition^2^ | 2 (6.1) |
| Maternal health indication | 3 (9.1) |
|  |  |
| Stillbirth evaluation components performed |  |
| Placental pathology | 27 (81.8) |
| Fetal autopsy | 12 (36.4) |
| Fetal genetic testing | 21 (63.6) |
|  |  |
| Fetal infectious testing performed |  |
| Bacterial culture | 3 (9.1) |
| Cytomegalovirus PCR | 6 (18.2) |
| Toxoplasma PCR | 0 (0) |
| Parvovirus PCR | 3 (9.1) |
|  |  |
| Maternal infectious testing performed |  |
| Cytomegalovirus IgG | 2 (6.1) |
| Cytomegalovirus IgM | 2 (6.1) |
| Toxoplasma IgM | 6 (18.2) |
| Parvovirus IgG | 1 (3.0) |
| Parvovirus IgM | 1 (3.0) |
| Syphilis^3^ | 30 (90.9) |

1: Multiple reasons were present in some cases

2: One with fetal findings due to cytomegalovirus infection, the other by human-herpesvirus 6

3: Includes treponemal and/or non-treponemal testing

**Supplemental Table 2: Characteristics of Stillbirths and Stillbirth Evaluations among Singleton vs Multiple Gestation Pregnancies**

| **Characteristic (n, % or median, IQR)** | **Total** | **Singleton Gestation** | **Multiple Gestation** | **p-value** |
| --- | --- | --- | --- | --- |
|  | **N=399** | **N=354** | **N=45** |  |
| **Maternal Medical History** |  |  |  |  |
| Maternal Age | 29.0 (24.0-34.0) | 29.0 (25.0-34.0) | 29.0 (24.0-34.0) | 0.90 |
| Maternal Race |  |  |  | 0.33 |
| Asian | 25 (6.3%) | 21 (5.9%) | 4 (8.9%) |  |
| Black | 215 (53.9%) | 197 (55.6%) | 18 (40.0%) |  |
| Native American or Alaskan Native | 2 (0.5%) | 2 (0.6%) | 0 (0.0%) |  |
| Native Hawaiian or Pacific Islander | 2 (0.5%) | 2 (0.6%) | 0 (0.0%) |  |
| Unknown | 56 (14.0%) | 47 (13.3%) | 9 (20.0%) |  |
| White | 99 (24.8%) | 85 (24.0%) | 14 (31.1%) |  |
| Diabetes | 33 (8.3%) | 20 (5.6%) | 13 (28.9%) | <0.001 |
| Chronic hypertension | 46 (11.5%) | 42 (11.9%) | 4 (8.9%) | 0.56 |
| Lupus | 4 (1.0%) | 3 (0.8%) | 1 (2.2%) | 0.38 |
| Other autoimmune disease | 9 (2.3%) | 7 (2.0%) | 2 (4.4%) | 0.27 |
| History of thromboembolism | 1 (0.3%) | 1 (0.3%) | 0 (0.0%) | 1.00 |
| Epilepsy | 1 (0.3%) | 1 (0.3%) | 0 (0.0%) | 1.00 |
| Anemia | 39 (9.8%) | 36 (10.2%) | 3 (6.7%) | 0.60 |
| Cardiac disease | 9 (2.3%) | 8 (2.3%) | 1 (2.2%) | 1.00 |
|  |  |  |  |  |
| **Obstetric History** |  |  |  |  |
| Recurrent spontaneous abortions | 44 (11.0%) | 40 (11.3%) | 4 (8.9%) | 0.80 |
| History of prior stillbirth | 27 (6.8%) | 25 (7.1%) | 2 (4.4%) | 0.75 |
| History of hereditary diagnosis | 10 (2.5%) | 9 (2.5%) | 1 (2.2%) | 1.00 |
| Consanguinity | 5 (1.3%) | 5 (1.4%) | 0 (0.0%) | 1.00 |
|  |  |  |  |  |
| **Complications of Current Pregnancy** |  |  |  |  |
| Any prenatal care | 371 (93.0%) | 329 (92.9%) | 42 (93.3%) | 1.00 |
| Preeclampsia | 64 (16.0%) | 59 (16.7%) | 5 (11.1%) | 0.40 |
| Placental abruption | 62 (15.5%) | 54 (15.3%) | 8 (17.8%) | 0.66 |
| Cholestasis of pregnancy | 0 (0.0%) | 0 (0.0%) | 0 (0.0%) |  |
| Respiratory infection during pregnancy | 28 (7.0%) | 26 (7.3%) | 2 (4.4%) | 0.76 |
| Genitourinary infection during pregnancy | 75 (18.8%) | 66 (18.6%) | 9 (20.0%) | 0.84 |
|  |  |  |  |  |
| **Delivery Characteristics** |  |  |  |  |
| Gestational Age (weeks) | 28.7 (23.0-35.1) | 29.6 (23.6-35.6) | 23.0 (21.7-31.4) | <0.001 |
| Cesarean Delivery | 56 (14.0%) | 45 (12.7%) | 11 (24.4%) | 0.041 |
| Obstetric diagnosis of chorioamnionitis | 57 (14.3%) | 50 (14.2%) | 7 (15.6%) | 0.82 |
|  |  |  |  |  |
| **Testing in Current Pregnancy** |  |  |  |  |
| Placental pathology | 387 (97.0%) | 342 (96.6%) | 45 (100.0%) | 0.38 |
| Documented gross fetal examination | 252 (63.2%) | 233 (65.8%) | 19 (42.2%) | 0.003 |
| Fetopsy | 126 (31.6%) | 113 (31.9%) | 13 (28.9%) | 0.74 |
| Any fetal genetic testing | 162 (40.6%) | 149 (42.1%) | 13 (28.9%) | 0.11 |
| Karyotype | 115 (28.8%) | 109 (30.8%) | 6 (13.3%) | 0.014 |
| Microarray | 115 (28.8%) | 103 (29.1%) | 12 (26.7%) | 0.86 |
| Bacterial culture from placenta or fetus | 73 (18.3%) | 68 (19.2%) | 5 (11.1%) | 0.22 |
| Cytomegalovirus PCR from placenta or fetus | 31 (7.8%) | 29 (8.2%) | 2 (4.4%) | 0.56 |
| Maternal cytomegalovirus IgG or IgM | 65 (16.3%) | 64 (18.1%) | 1 (2.2%) | 0.004 |
| Toxoplasmosis PCR from placenta or fetus | 4 (1.0%) | 4 (1.1%) | 0 (0.0%) | 1.00 |
| Maternal toxoplasmosis IgM | 57 (14.3%) | 52 (14.7%) | 5 (11.1%) | 0.65 |
| Parvovirus PCR from placenta or fetus | 13 (3.3%) | 13 (3.7%) | 0 (0.0%) | 0.38 |
| Maternal parvovirus IgG or IgM | 148 (37.1%) | 146 (41.2%) | 2 (4.4%) | <0.001 |
| Antiphospholipid antibody testing | 222 (55.6%) | 217 (61.3%) | 5 (11.1%) | <0.001 |

**Supplemental Table 3: Completion of Stillbirth Evaluation Components, Pre- (2017-2019) and Post-Guideline Publication (2020-2022)**

|  | **Stillbirths Pre-Guideline Publication** | **Stillbirths Post-Guideline Publication** | **p-value** |
| --- | --- | --- | --- |
|  | n (%) | |  |
| **Fetal-Placental Evaluation** | **N = 198** | **N = 201** |  |
| Placental pathology | 193 (97.4) | 194 (96.5) | 0.58 |
| Documented gross fetal  examination | 128 (64.7) | 124 (61.7) | 0.54 |
| Genetic analysis | 68 (34.3) | 94 (46.8) | 0.01 |
| Fetal autopsy | 55 (27.8) | 71 (35.3) | 0.11 |
|  |  |  |  |
| **Fetal Infectious Testing** |  |  |  |
| Bacterial culture | 39 (19.7) | 34 (16.9) | 0.47 |
| Cytomegalovirus PCR | 16 (8.1) | 15 (7.5) | 0.82 |
| Parvovirus PCR | 8 (4.0) | 5 (2.5) | 0.38 |
| Toxoplasma PCR | 4 (2.0) | 0 (0) | 0.04 |
|  |  |  |  |
| **Maternal Evaluation** | **N = 195** | **N = 194** |  |
| Antiphospholipid antibody  testing^1^ | 103 (52.8) | 118 (60.8) | 0.11 |
| Kleihauer-Betke testing | 99 (51.3) | 97 (50.0) | 0.88 |
| Toxicology screen | 100 (51.3) | 96 (49.5) | 0.72 |
| Glucose tolerance testing  available^2^ | 68 (34.3) | 94 (46.8) | 0.01 |
| Hemoglobin A1c | 147 (75.4) | 150 (77.3) | 0.65 |
|  |  |  |  |
| **Maternal Infectious Testing** |  |  |  |
| Syphilis^3^ | 189 (96.9) | 186 (95.9) | 0.58 |
| Parvovirus IgG/IgM | 38 (19.5) | 44 (22.7) | 0.44 |
| Cytomegalovirus IgG/IgM | 31 (15.9) | 34 (17.5) | 0.67 |
| Toxoplasma IgM | 25 (12.8) | 31 (16.0) | 0.38 |

1: Includes lupus anticoagulant, anticardiolipin antibodies, and/or beta-2 glycoprotein antibodies

2: At minimum, available results of 1-hour glucose tolerance testing

3: Includes treponemal and non-treponemal testing

**Supplemental Table 4: Stillbirth Evaluation Components, by GA category**

|  | **20-23 weeks GA**  N = 120 | **24-27 weeks GA**  N = 69 | **28-33 weeks GA**  N = 83 | **34-36 weeks GA**  N = 66 | **≥37 weeks GA**  N = 55 | **p-value** |
| --- | --- | --- | --- | --- | --- | --- |
| **Component** | n (%) | | | | |  |
| Placental pathology | 117 (98) | 67 (97) | 80 (96) | 65 (98) | 53 (96) | 0.94 |
| Fetal autopsy | 20 (17) | 24 (35) | 34 (41) | 24 (36) | 23 (42) | 0.001* |
| Fetal genetics | 33 (28) | 41 (59) | 36 (43) | 23 (35) | 27 (49) | <0.001 |

*Linear trend demonstrated by Cochrane-Armitage test, p<0.05
